# Glycoprotein nonmetastatic melanoma protein B regulates lysosomal integrity and lifespan of senescent cells

**DOI:** 10.1038/s41598-022-10522-3

**Published:** 2022-04-20

**Authors:** Masayoshi Suda, Ippei Shimizu, Goro Katsuumi, Chieh Lun Hsiao, Yohko Yoshida, Naomi Matsumoto, Yutaka Yoshida, Akihiro Katayama, Jun Wada, Masahide Seki, Yutaka Suzuki, Shujiro Okuda, Kazuyuki Ozaki, Mayumi Nakanishi-Matsui, Tohru Minamino

**Affiliations:** 1 Department of Cardiovascular Biology and Medicine, Juntendo University Graduate School of Medicine, 2-1-1 Hongo, Bunkyo-ku, Tokyo, 113-8421 Japan; 2 Department of Advanced Senotherapeautics, Juntendo University Graduate School of Medicine, Tokyo, 113-8421 Japan; 3 Division of Biochemistry, School of Pharmacy, Iwate Medical University, Iwate, 028-3694 Japan; 4 Department of Structural Pathology, Kidney Research Center, Niigata University Graduate School of Medical and Dental Sciences, Niigata, 951-8510 Japan; 5 Department of Nephrology, Rheumatology, Endocrinology and Metabolism, Dentistry and Pharmaceutical Sciences, Okayama University Graduate School of Medicine, Okayama, 700-8558 Japan; 6 Department of Computational Biology and Medical Sciences, Graduate School of Frontier Sciences, The University of Tokyo, Chiba, 277-8561 Japan; 7 Division of Bioinformatics, Niigata University Graduate School of Medical and Dental Sciences, Niigata, 951-8510 Japan; 8 Department of Cardiovascular Biology and Medicine, Niigata University Graduate School of Medical and Dental Sciences, Niigata, 951-8510 Japan; 9 Japan Agency for Medical Research and Development-Core Research for Evolutionary Medical Science and Technology (AMED-CREST), Japan Agency for Medical Research and Development, Tokyo, Japan

**Keywords:** Cardiovascular biology, Experimental models of disease

## Abstract

Accumulation of senescent cells in various tissues has been reported to have a pathological role in age-associated diseases. Elimination of senescent cells (senolysis) was recently reported to reversibly improve pathological aging phenotypes without increasing rates of cancer. We previously identified glycoprotein nonmetastatic melanoma protein B (GPNMB) as a seno-antigen specifically expressed by senescent human vascular endothelial cells and demonstrated that vaccination against Gpnmb eliminated Gpnmb-positive senescent cells, leading to an improvement of age-associated pathologies in mice. The aim of this study was to elucidate whether GPNMB plays a role in senescent cells. We examined the potential role of GPNMB in senescent cells by testing the effects of GPNMB depletion and overexpression in vitro and in vivo. Depletion of GPNMB from human vascular endothelial cells shortened their replicative lifespan and increased the expression of negative cell cycle regulators. Conversely, GPNMB overexpression protected these cells against stress-induced premature senescence. Depletion of Gpnmb led to impairment of vascular function and enhanced atherogenesis in mice, whereas overexpression attenuated dietary vascular dysfunction and atherogenesis. GPNMB was upregulated by lysosomal stress associated with cellular senescence and was a crucial protective factor in maintaining lysosomal integrity. GPNMB is a seno-antigen that acts as a survival factor in senescent cells, suggesting that targeting seno-antigens such as GPNMB may be a novel strategy for senolytic treatments.

## Introduction

Previous studies demonstrated that senescent cells accumulate in various tissues with aging or in response to metabolic stress^1,2^. Accumulation of senescent cells in tissues has been suggested to contribute to age-associated pathologies. For example, senescent vascular cells are observed in human and mouse atherosclerotic plaques^3–5^, and inhibiting senescence of vascular cells by deleting negative cell cycle regulators such as p53 or p21 led to improvement of vascular dysfunction in diabetic mice^6,7^ and to amelioration of atherogenesis in atherosclerosis-prone mice^8^. Likewise, accumulation of senescent cells was observed in visceral adipose tissues of diabetic patients and mice and was found to contribute to insulin resistance and glucose intolerance^9^. Inhibition of p53 in adipose tissues was shown to improve metabolic abnormalities associated with dietary obesity by inhibiting production of inflammatory molecules known as senescence-associated secretary phenotype (SASP) factors from senescent adipose tissues^9–11^.

Recently, a mouse model with genetic elimination of senescent cells was established, and eliminating senescent cells (senolysis) in this model was found to reversibly improve pathological aging and extend the lifespan without increasing the risk of cancer^12–19^. More recently, several senolytic agents were shown to reversibly improve pathological aging and extend the healthy lifespan in aged mice^20–25^. In a previous study^26^, we took another approach to senolytic therapy that was based on targeting seno-antigens specifically expressed by senescent cells. We used transcriptome data from senescent human vascular endothelial cells to search for molecules with transmembrane domains and thereby identified glycoprotein nonmetastatic melanoma protein B (GPNMB),^27–30^ which is expressed by human vascular endothelial cells, as a candidate seno-antigen. GPNMB expression was significantly upregulated in vascular endothelial cells of patients and mice with atherosclerosis. Elimination of Gpnmb-positive senescent cells by vaccination improved age-associated pathologies, including atherosclerosis and metabolic abnormalities, and extended lifespan of mice with premature aging, suggesting that vaccination targeting seno-antigens could be a potential strategy for novel senolytic therapy. However, little is known about the role of GPNMB in cellular senescence. Therefore, in the current study we sought to investigate the molecular mechanisms underlying the regulation of GPNMB expression and elucidate the potential role of GPNMB in senescent vascular endothelial cells.

## Results

### GPNMB positively regulates cellular lifespan

**Figure 1:**
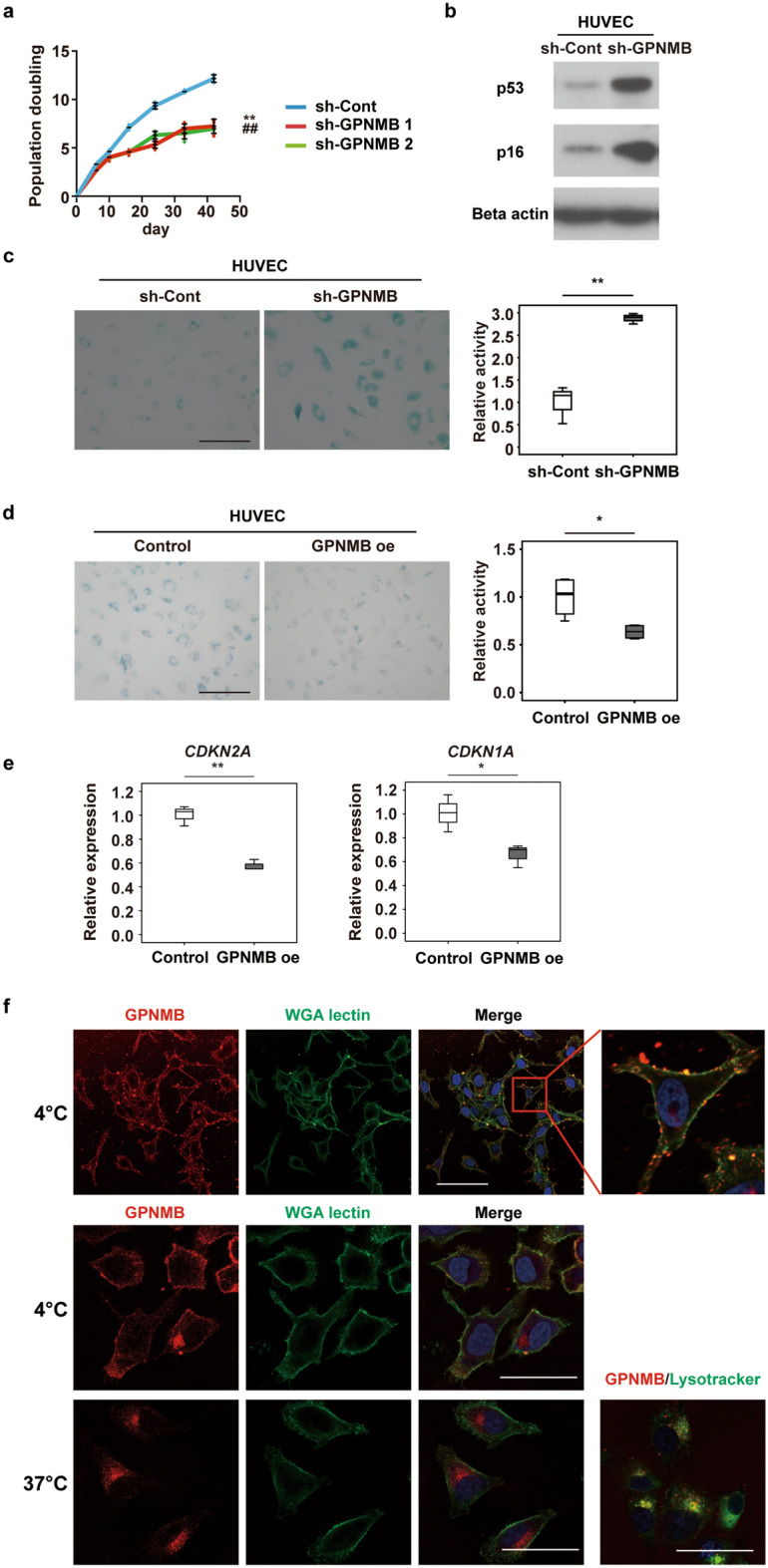
GPNMB positively regulates cellular lifespan. (**a**) Replicative lifespan of HUVECs infected with a retroviral vector expressing shRNAs targeting *GPNMB* (sh-GPNMB1 or sh-GPNMB2) or a control vector (sh-Cont). Infected cells were passaged until the termination of replication and the number of population doublings was determined (n = 3 for sh-Cont, n = 4 for sh-GPNMB1, and n = 4 for sh-GPNMB2). **P < 0.01, sh-Cont vs. sh-GPNMB1; ##*P* < 0.01, sh-Cont vs. sh-GPNMB2. (**b)** Western blot analysis of p53 and p16 expression by HUVECs infected with a retroviral vector expressing shRNA for GPNMB (sh-GPNMB) or a control vector (sh-Cont). Original blots are presented in Supplementary Fig. 6. (**c)** SA-β-gal assay of HUVECs infected with a retroviral vector expressing shRNA for GPNMB (sh-GPNMB) or a control vector (sh-Cont) at passage 7. Representative photomicrographs are shown at × 200 magnification. Scale bar = 200 μm. The right graph displays quantification of SA-β-gal activity (n = 3 each). (**d,e)** SA-β-gal assay and PCR analysis of doxorubicin-induced senescent HUVECs infected with a GPNMB overexpression vector (GPNMB oe) or a control vector (Control) ((**d)** n = 4 for each; **e,** n = 3 for each). Representative photomicrographs are shown. Scale bar = 200 μm. The graphs display quantification of SA-β-gal activity (**d**) and expression of senescence makers (**e**). (**f)** Immunostaining for GPNMB in SK-MEL-2 at 4 °C and 37 °C. Plasma membrane was stained with WGA lectin (green), nuclei were labeled with Hoechst (blue), and lysosomes were labeled with Lysotracker (green). Scale bar (upper panel) = 100 μm. Scale bar (middle and lower panels) = 50 μm. The data were analyzed by repeated measures analysis followed by Tukey’s multiple comparison test (**a**) or by the two-tailed Student’s *t* test (**c–e**). *P < 0.05, **P < 0.01. The data are shown as the mean ± SD with plots of all individual data (**a**) or box and whisker plots (**c–e**).

To investigate the role of GPNMB in cellular senescence, we examined the effects of short hairpin RNA targeting GPNMB (sh-GPNMB) on the replicative lifespan of human vascular endothelial cells. Infection of human vascular endothelial cells with a retroviral vector encoding sh-GPNMB caused depletion of GPNMB and shortened the replicative lifespan of these cells, along with an increase in the expression of p53 and p16^Ink4a^, as well as elevation of the activity of senescence-associated β-galactosidase (SA-β-gal) (Fig. 1a–c). Conversely, overexpression of GPNMB inhibited premature senescence of human vascular endothelial cells induced by doxorubicin (Fig. 1d,e and Supplementary Fig. 1a–g), suggesting that GPNMB protected these cells against senescence. To analyze the cellular localization of GPNMB, we conducted immunostaining for GPNMB in a melanoma cell line that expresses high levels of GPNMB. We found that GPNMB immunoreactivity could be detected in the plasma membrane at 4 °C. When cells were maintained at 37 °C, GPNMB was subsequently internalized and localized in lysosomes (Fig. 1f).

### GPNMB plays a critical role in lysosomal integrity

**Figure 2:**
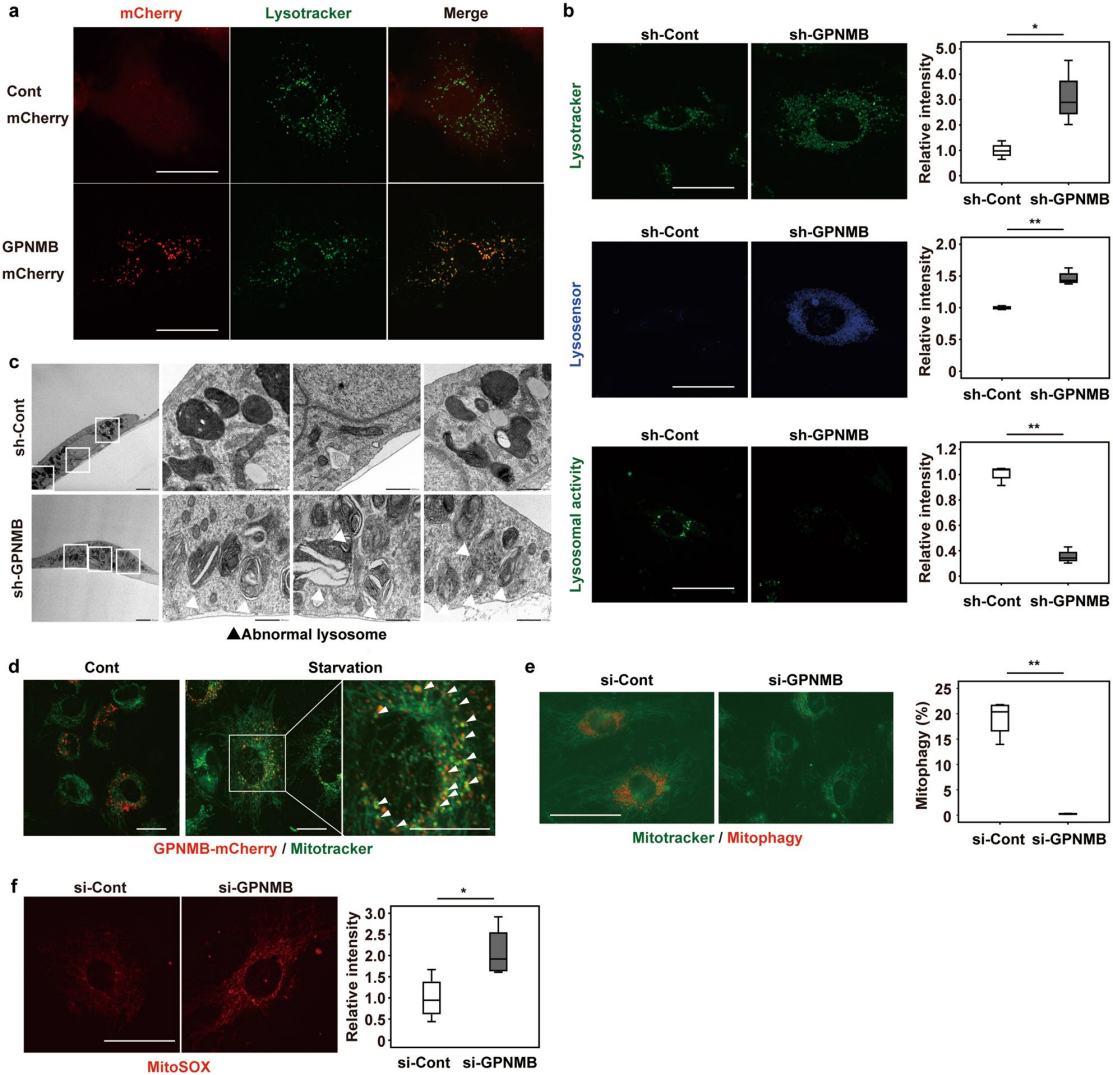
GPNMB plays a critical role in lysosomal integrity. (**a)** Fluorescent imaging of GPNMB-mCherry (red) and lysosomes (LysoTracker Green; green). Scale bar = 25 μm. (**b)** Fluorescent imaging of lysosomes (LysoTracker Green; green), lysosomal pH (Lysosensor; blue), and lysosomal enzymatic activity (Lysosomal Intracellular Activity Assay; green) in HUVECs infected with a retroviral vector expressing shRNA for GPNMB (sh-GPNMB) or a control vector (sh-Cont). The right graphs display quantification of staining intensity (n = 3 each). Scale bar = 25 μm. (**c)** Transmission electron microscopy of HUVECs infected with a retroviral vector expressing shRNA for GPNMB (sh-GPNMB) or a control vector (sh-Cont). Scale bar = 2 μm for low-magnification images and 500 nm for high-magnification images. (**d)** Fluorescent imaging of GPNMB-mCherry (red) and mitochondria (MitoTracker; green) in HUVECs incubated for 8 h with (Starvation) or without (Cont) starvation for amino acids and serum. White arrow heads indicate co-localization of GPNMB expression with mitochondria. Scale bar = 20 μm. (**e)** Fluorescent imaging of mitophagy (Mitophagy dye; red) and mitochondria (MitoTracker; green), showing that introduction of siRNA for GPNMB (si-GPNMB) impairs mitophagy in senescent HUVECs after starvation for 8 h compared to cells infected with control siRNA (si-Cont). The right graph displays quantification of staining intensity (n = 4 each). Scale bar = 50 μm. (**f)** Fluorescent imaging of reactive oxygen species in mitochondria (MitoSox) in HUVECs after introduction of si-GPNMB or si-Cont. The right panel displays quantification of staining intensity (n = 4 each). Scale bar = 25 μm. The data were analyzed by the two-tailed Student’s *t* test and are presented as box and whisker plots (**b**,**e**,**f**). *P < 0.05, **P < 0.01.

To further assess the role of GPNMB in senescent vascular endothelial cells, we investigated its localization in human vascular endothelial cells overexpressing GPNMB-mCherry protein. Fluorescent imaging revealed that GPNMB was co-localized with Lysotracker, suggesting a role in the lysosomes (Fig. 2a), which was similar to that in the melanoma cell line. Immunoelectron microscopy in human vascular endothelial cells overexpressing GPNMB-mCherry protein also demonstrated localization of GPNMB in the lysosomes, as well as in the plasma membrane (Supplementary Fig. 2). We next assessed the number and function of lysosomes in vascular endothelial cells with GPNMB knockdown. Depletion of GPNMB resulted in an increase of lysosomes (Fig. 2b, upper), but lysosomal enzyme activity decreased along with marked elevation of the pH (Fig. 2b, middle and bottom), suggesting that GPNMB was a positive regulator of lysosome function. Electron microscopy revealed accumulation of dysfunctional lysosomes in GPMNB-depleted vascular endothelial cells (Fig. 2c). We found that GPNMB expression was co-localized with the mitochondria after starvation, suggesting a potential role in the process of mitophagy (Fig. 2d). Further investigation demonstrated that GPNMB depletion induced lysosomal dysfunction, leading to impairment of the mitophagic response to starvation and accumulation of reactive oxygen species in the mitochondria (Fig. 2e,f).

### A potential role of GPNMB in lysosomal V-ATPase

**Figure 3:**
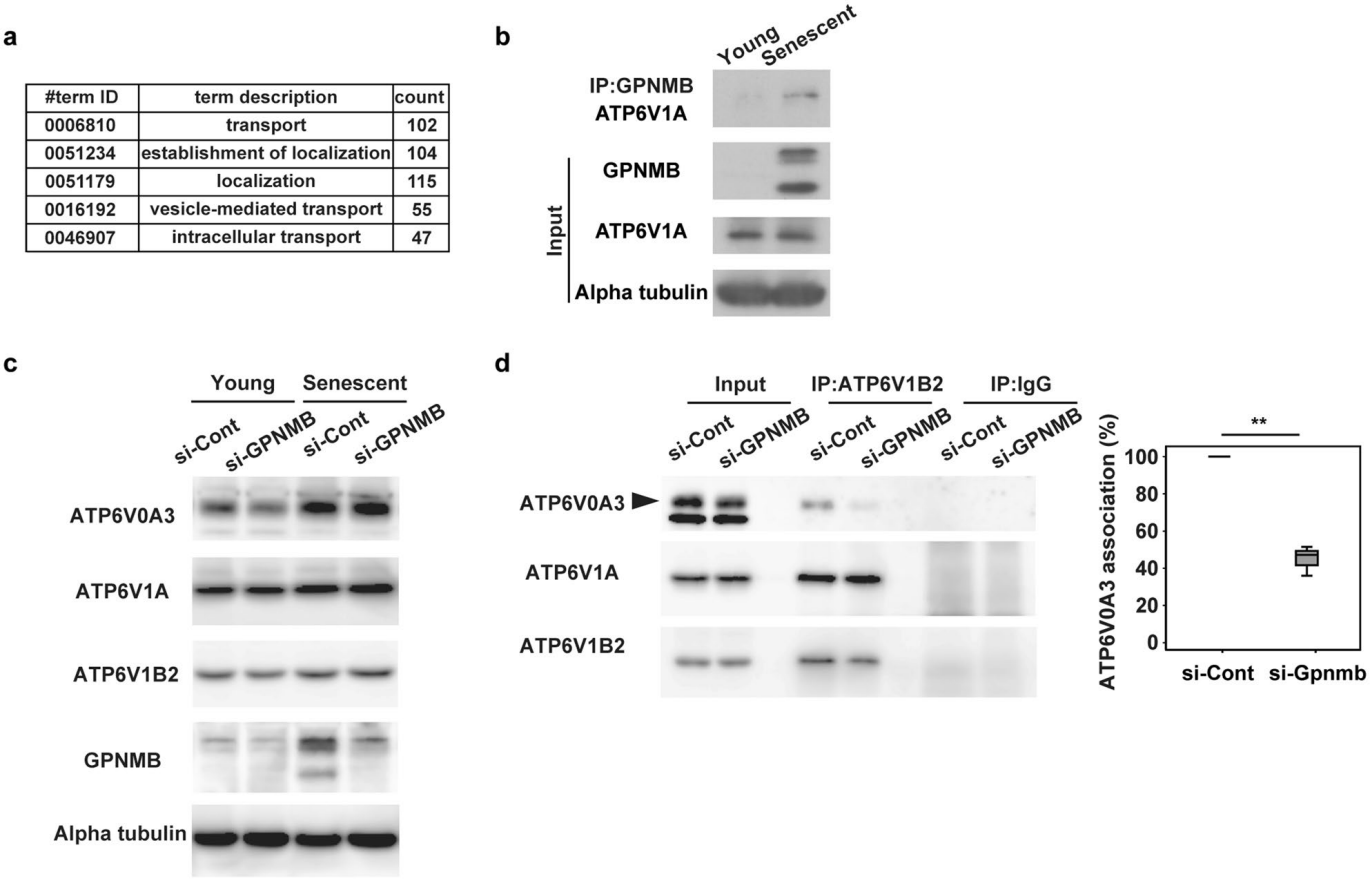
GPNMB plays a critical role in lysosomal integrity. (**a)** GO terms for GPNMB-binding proteins identified by LC–MS/MS. (**b)** Binding of GPNMB to ATP6V1A in young and senescent HUVECs by immunoprecipitation (IP) with anti-GPNMB antibody and Western blot with anti-ATP6V1A antibody. Original blots are presented in Supplementary Fig. 7. (**c)** Expression of ATP6V0A3, ATP6V1A, and ATP6V1B2 in young and senescent HUVECs following treatment with si-GPNMB or si-Cont. Original blots are presented in Supplementary Fig. 8. (**d)** Association between the V_o_ domain and the V_1_ domain in senescent HUVECs treated with si-GPNMB or si-Cont by IP with anti-ATP6V1B2 antibody and Western blot with anti-ATP6V0A3 antibody, anti-ATP6V1A antibody, or anti-ATP6V1B2 antibody. Original blots are presented in Supplementary Fig. 9. The graph on the right shows the quantification of ATP6V0A3 associated with ATP6V1B2 (n = 3) calculated by the following formula: IP si-GPNMB ATP6V0A3/INPUT si-GPNMB ATP6V0A3 vs. IP si-Cont ATP6V0A3/INPUT si-Cont ATP6V0A3. The data were analyzed by the two-tailed Student’s *t* test and are presented as box and whisker plots (**d**). *P < 0.05, **P < 0.01.

It has been reported that GPNMB may interact with several molecules to participate in various cellular functions, including maintenance of lysosomal integrity^31^. We therefore conducted immunoprecipitation studies of human vascular endothelial cells overexpressing GPNMB-mCherry protein or mCherry protein using an anti-mCherry antibody and subjected the cell precipitates to LC–MS/MS analysis. Consistent with a previous report^32^, this analysis revealed binding of GPMNB to various lysosomal components (including V-type ATPase), as well as binding to components of the endosomal, mitochondrial, and plasma membrane fractions (Fig. 3a and Supplementary Table 1). Western blot analysis of immunoprecipitates using an anti-GPNMB antibody confirmed binding of GPNMB to V-type ATPase in senescent endothelial cells (Fig. 3b), further supporting a role of GPNMB in lysosomal function.

The V-type ATPases are large multi-subunit complexes organized into two domains operating by a rotary mechanism^33^. The V_1_ domain, a peripheral complex located on the cytoplasmic side of the membrane, performs ATP hydrolysis. The V_o_ domain is a membrane-embedded complex, responsible for the translocation of protons from the cytoplasm to the lumen or extracellular space. It has been demonstrated that the V-type ATPase activity is controlled by regulation of the assembly of the enzyme complex, in particular, the reversible dissociation of the V_1_ domains and V_o_ domains^34–36^. In the current study, it was hypothesized that GPNMB could control V-type ATPase activity by regulating the binding between the V_1_ and V_o_ domains. Expression of the V_1_ domains (such as ATP6V1A) in senescent endothelial cells was increased or unchanged compared with non-senescent endothelial cells (Fig. 3c and Supplementary Fig. 3). Likewise, expression of the V_o_ domains (such as ATP6V0A3) in senescent endothelial cells was increased or unchanged compared with non-senescent endothelial cells (Fig. 3c and Supplementary Fig. 2). Expression of V_1_ or V_o_ domains was not affected by deletion of GPNMB (Fig. 3c). In contrast, deletion of GPNMB significantly dissociated the V_1_ and V_o_ domains in senescent endothelial cells (Fig. 3d). These results suggest that GPNMB may regulate the binding between the V_1_ and V_o_ domains and that this regulation may affect V-type ATPase activity.

### GPNMB expression is upregulated by lysosomal stress

**Figure 4:**
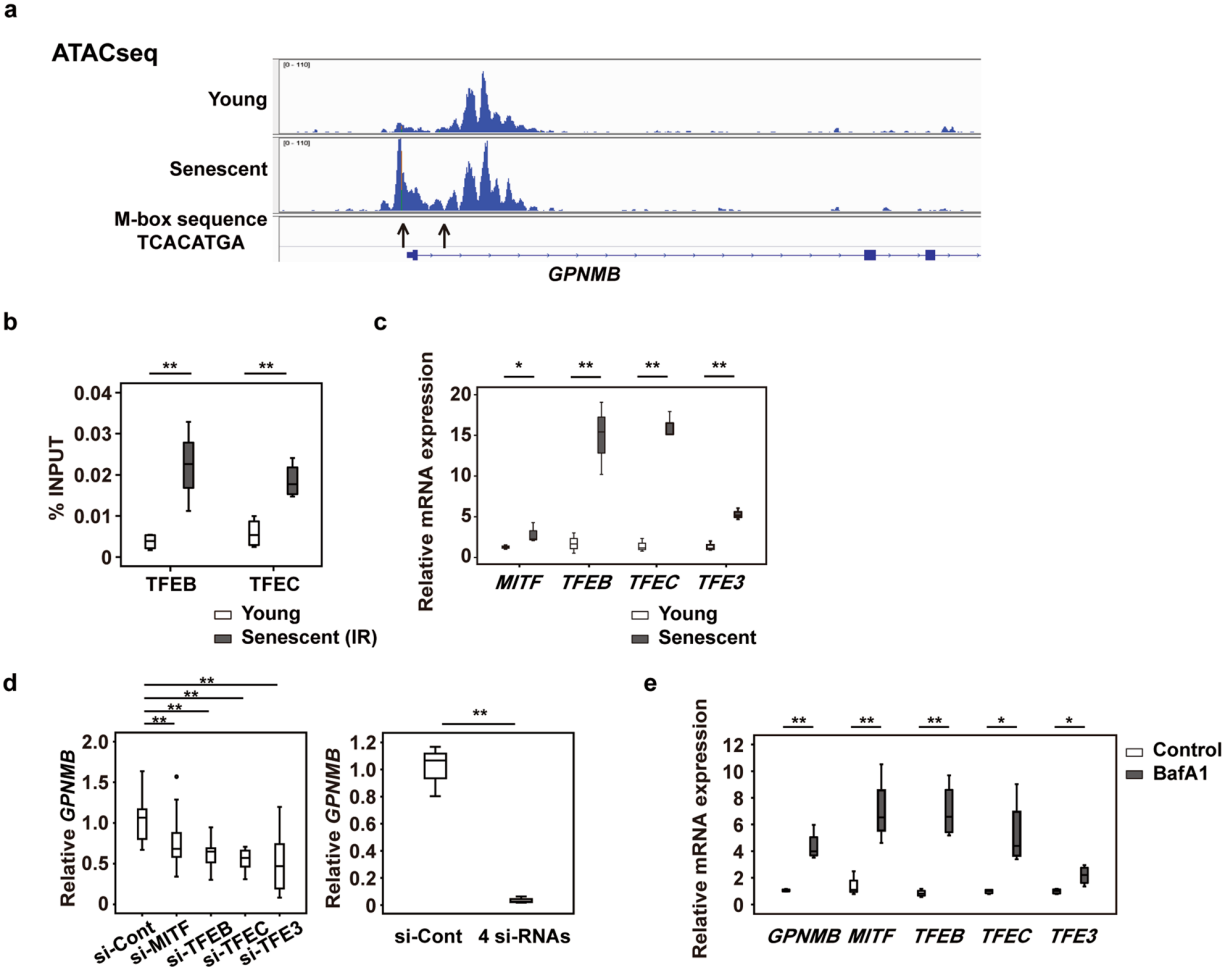
GPNMB expression is upregulated by lysosomal stress. (**a)** ATAC-seq of HUVECs. (**b)** ChIP-qPCR analysis of TFEB and TFEC transcription factors in young HUVECs and irradiation-induced senescent HUVECs (n = 4 each). The 10% of sonicated sample was used as an input control. The ratio of precipitated chromatin (%INPUT) was calculated as follows: %INPUT = 2^(Ct (INPUT) – log^_2_^10 – Ct (IP))^*100. (**c)** Relative expression of the MITF/TFE transcription factors in young and replicative senescent HUVECs examined by qPCR (n = 3 each). (**d)** The left graph displays relative expression of *GPNMB* in replicative senescent HUVECs after introduction of siRNAs for the MITF/TFE transcription factors (si-MITF, si-TFEB, si-TFEC, and si-TFE3) or control siRNA (si-Cont) (n = 6 for si-Cont, n = 8 for si-MITF, n = 8 for si-TFEB, n = 8 for si-TFEC, and n = 8 for si-TFE3). Two outliers (○) were detected in the si-MITF and si-TFEB group by boxplot analysis and were excluded from statistical analysis. The right graph displays relative expression of *GPNMB* after introduction of a mixture of 4 siRNAs (si-MITF, si-TFEB, si-TFEC, and si-TFE3) or control siRNA (si-Cont). (n = 3 each). **e,** qPCR showing relative expression of *GPNMB* and the transcription factors MITF/TFEs in HUVECs treated for 24 h with 100 nmol l^–1^ bafilomycin A1 (BafA1, n = 4) or the vehicle (Control, n = 4). The data were analyzed by the two-tailed Student’s *t* test (**b**–**e**), or by one-way ANOVA followed by Tukey’s multiple comparison test (**d**). *P < 0.05, **P < 0.01. The data are shown as box and whisker plots (**b**–**e**).

To elucidate the mechanisms regulating GPNMB expression, we conducted the Assay for Transposase-Accessible Chromatin Sequencing (ATAC-seq) in human vascular endothelial cells. The results indicated that members of the microphthalmia family involved in lysosomal biogenesis^37^, such as microphthalmia-associated transcription factor (MITF), transcription factor EB (TFEB), TFE3, and TFEC, were potentially regulators of GPNMB expression (Fig. 4a). Chromatin immunoprecipitation (ChIP-qPCR) detected significant enrichment of MITF/TFE transcription factors at the response element of the *GPNMB* locus in senescent vascular endothelial cells (Fig. 4b). Consistent with these results, MITF/TFE transcription factors were upregulated in senescent vascular endothelial cells (Fig. 4c). Depleting one of these transcription factors led to a significant decrease of *GPNMB* expression by senescent vascular endothelial cells, while combined depletion of all four factors caused further downregulation of *GPNMB* expression (Fig. 4d, Supplementary Fig. 4). Conversely, induction of lysosomal stress by bafilomycin A1 upregulated the expression of these transcription factors and also increased *GPNMB* expression (Fig. 4e). These results suggested that lysosomal stress associated with cellular senescence leads to upregulation of MITF/TFE transcription factors and thus increases GPNMB expression. Augmentation of GPNMB expression may improve lysosomal function and mitophagy, thereby protecting cells against a senescence-induced increase of lysosomal stress. However, it remains to be determined which member of the MITF/TFE family substantially contributes to expression of GPNMB in senescent vascular endothelial cells.

### Mouse models of Gpnmb deficiency and Gpnmb overexpression

**Figure 5:**
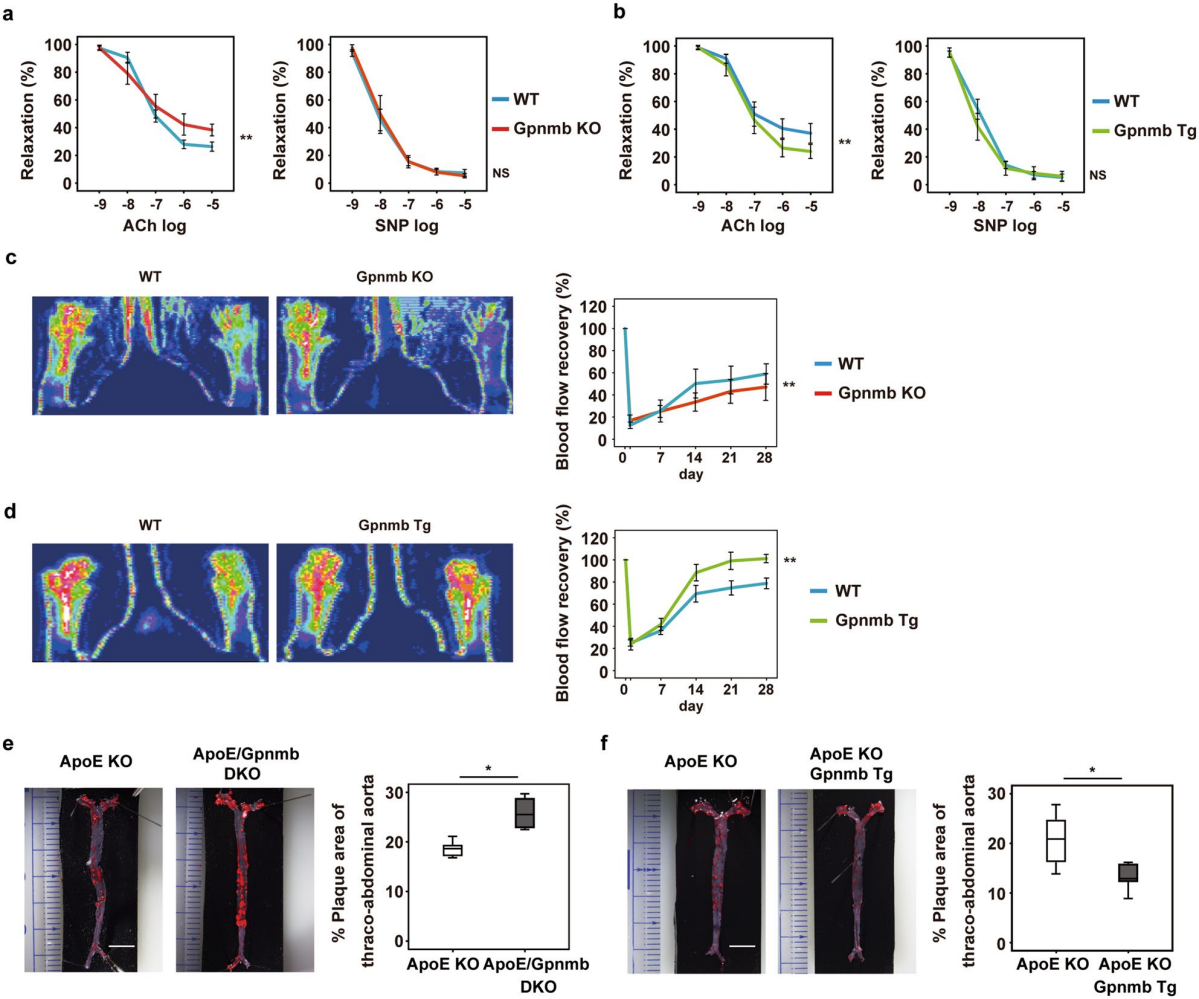
Gpnmb has a protective effect against vascular pathology. **(a**,**b)** Endothelium-dependent (left) and endothelium-independent (right) vasorelaxation were examined in the iliac arteries of Gpnmb knockout (Gpnmb KO) mice and littermate controls (WT) after feeding an HFD for 8 weeks (n = 9 each) (**a**), or in arteries from Gpnmb-overexpressing transgenic (Gpnmb Tg) mice and littermate controls (WT) after feeding the HFD for 8 weeks (n = 6 each) (**b**). (**c**,**d)** Laser Doppler perfusion imaging of hind limbs from Gpnmb KO mice and littermate controls (WT) (**c**), or Gpnmb Tg mice and littermate controls (WT), after 14 days of ischemia (**d**). The right graphs show quantification of blood flow recovery on Laser Doppler perfusion imaging at the indicated times (**c**, n = 9 each; **d**, n = 9 for WT and n = 6 for Tg). (**e**,**f)** Oil red O staining of thoraco-abdominal aortas from ApoE KO mice and ApoE KO/Gpnmb KO (DKO) mice (**e**), or ApoE KO mice and ApoE KO/Gpnmb Tg mice (**f**). The right graphs display quantification of the plaque area in the thoraco-abdominal aorta (**e**, n = 6 for ApoE KO and n = 4 for ApoE KO/Gpnmb KO; **f**, n = 6 each). Data were analyzed by repeated measures analysis followed by Tukey’s multiple comparison test (**a**–**d**), or by the two-tailed Student’s *t* test (**e** and **f**). *P < 0.05, **P < 0.01, NS (not significant). The data are shown as the mean ± SEM with plots of all individual data (**a–d**) or box and whisker plots (**e**,**f**).

Next, we examined the effects of Gpnmb knockout (KO) or Gpnmb overexpression in mice. DBA/2 J mice (with a C57BL/6 background) expressing a stop codon mutation of Gpnmb (R150X)^29^ were used as the Gpnmb KO model, while *Fabp4* promoter-driven Gpnmb transgenic mice^38^ overexpressing Gpnmb in adipocytes, endothelial cells, and macrophages were used as the Gpnmb overexpression model (Supplementary Fig. 5a). Mice from both groups were fed an HFD and we examined vascular function. Ablation of Gpnmb led to impairment of endothelium-dependent vasodilatation, whereas Gpnmb overexpression resulted in improvement of dietary vascular dysfunction (Fig. 5a,b). In both models, there was no significant impairment of vascular smooth muscle relaxation (Fig. 5a,b).

We subsequently examined the role of Gpnmb in angiogenesis of ischemic tissues. We created hind limb ischemia in Gpnmb KO mice and Gpnmb overexpressing mice, and used a laser Doppler perfusion analyzer to assess blood flow recovery. We found that blood flow recovery in the ischemic limbs was significantly impaired by deletion of Gpnmb (Fig. 5c). In contrast, blood flow recovery was enhanced in the mice with Gpnmb overexpression (Fig. 5d), indicating that Gpnmb was a positive regulator of angiogenesis in ischemic tissues.

To further analyze the role of Gpnmb in vascular pathology, we generated ApoE /Gpnmb double KO mice by breeding DBA/2J mice with ApoE KO mice. We also generated ApoE KO mice with Gpnmb overexpression by breeding ApoE KO mice with *Fabp4* promoter-driven Gpnmb transgenic mice. Both of these mouse models were fed the HFD. We found that Gpnmb deletion promoted the formation of atherosclerotic plaque, while Gpnmb overexpression was associated with less plaque (Fig. 5e,f). These results suggested that GPNMB protects against stress, particularly in senescent cells. Consistent with this concept, HFD-induced upregulation of negative cell cycle regulators was attenuated by Gpnmb overexpression and augmented by Gpnmb deletion (Supplementary Fig. 5b).

## Discussion

We demonstrated that depletion of GPNMB enhanced senescence phenotypes of vascular cells, whereas overexpression of GPNMB attenuated pathological aging phenotypes of vascular cells in vitro and in vivo. GPNMB was upregulated by lysosomal stress associated with cellular senescence and played a protective role in maintaining lysosomal integrity. These results suggest that GPNMB may serve as a survival factor for senescent cells.

GPNMB is a type I transmembrane protein that was originally identified in melanoma cell lines and is preferentially expressed by non-metastatic cell lines, suggesting that it may inhibit tumorigenesis^39^. However, it was subsequently shown that Gpnmb is expressed on the surface of various malignant cells, including melanoma, glioblastoma, and triple-negative breast cancer, and functions as a tumor promoter^40^. Accordingly, an antibody–drug conjugate targeting GPNMB was developed and tested for various malignancies in phase I/II studies^41–43^. GPNMB has also been identified as osteoactivin in osteoblasts^27^, as well as being isolated from dendritic cells as DC-HIL, a dendritic cell-associated transmembrane protein^44^. Furthermore, it has been suggested that GPNMB may be involved in neurodegenerative disease^45,46^, as well as in cardiovascular and metabolic disease^38,47^. In addition, it was reported that GPNMB expressed on antigen-presenting cells negatively regulates T cell activation by binding to syndecan 4^30,48^. In the previous study^26^, we found that Gpnmb vaccination eliminated Gpnmb-positive senescent cells, but also senescent cells expressing lower levels of Gpnmb. Because it is assumed that senescent cells progressively accumulate in aged tissues by escaping from the endogenous senolytic immune system, our results suggest that targeting Gpnmb may enhance the endogenous senolytic immune system to promote elimination of senescent cells and suppress pathological aging.

Our results suggested that GPNMB was upregulated by lysosomal stress associated with cellular senescence, and acted as a protective factor for senescent cells. Consistent with this notion, deletion of GPNMB from young cells accelerated replicative senescence, whereas overexpression of GPNMB provided protection against premature senescence. While overexpression of GPNMB is potentially another strategy for preventing the accumulation of senescent cells, it has a risk of promoting of cancer. Thus, elimination of GPNMB-positive cells seems to be a more feasible strategy for clinical application. Upregulation of GPNMB has been reported in patients with inherited lysosomal storage disorders^49,50^, indicating that senescent cells with high expression of GPNMB may be affected by severe lysosomal stress and could be critically involved in organ aging via secretion of SASP factors and oxidants. Upregulation of SA-β-gal activity is a surrogate maker for senescent cells, suggesting that increased lysosomal stress may play an important role in induction of age-related pathology by senescent cells, and targeting lysosomal components like GPNMB could have a more specific senolytic effect.

In conclusion, our results taken together with the above considerations demonstrated that GPNMB was upregulated by lysosomal stress associated with cellular senescence and acted as a protective factor for senescent cells, and suggest that targeting cell/tissue-specific seno-antigens like GPNMB could be a reasonable strategy for next-generation senolytic therapy with higher selectivity and fewer off-target effects.

## Methods

### Animal models

All animal experiments were conducted in compliance with the guidelines which was reviewed by the Institutional Animal Care and Use Committee of Niigata University and Juntendo University, and this study is approved by the Institutional Animal Care and Use Committee of Niigata University and Juntendo University. The study was carried out in compliance with the ARRIVE guidelines. C57BL/6 mice were purchased from SLC Japan (Shizuoka, Japan), and were maintained on a high fat diet (HFD; CLEA) or normal chow (NC) from 4 to 12 weeks of age, unless otherwise described in the figure legends. DBA/2J and DBA/2 J-Gpnmb^+^ mice were obtained from the Jackson Laboratory. DBA/2J mice have a Gpnmb stop codon mutation (R150X)^29^and were used to generate the Gpnmb knockout (KO) model. Male DBA/2J mice were mated with C57BL/6 mice to obtain Gpnmb^+/−^ mice, and then were backcrossed with C57BL/6 mice for more than six generations. DBA/2J-Gpnmb^+^ mice with a C57BL/6 background were used as the control. Genotyping of Gpnmb^–/–^ mice was performed as described previously^38^. Gpnmb overexpressing transgenic (Tg) mice with a C57BL/6 background were generated and genotyped as described previously^38^. ApoE KO mice (C57BL/6 background) were obtained from the Jackson Laboratory and were maintained on a second HFD (Oriental Yeast Co., Ltd.) from 4 to 16 weeks of age. ApoE/Gpnmb KO mice (C57BL/6 background) and ApoE KO/DBA/2J-Gpnmb^+^ mice (C57BL/6 background) were generated by crossing ApoE KO mice with DBA/2J (Gpnmb^–/–^) or DBA/2J-Gpnmb^+^ mice, respectively, and were maintained on the second HFD from 4 to 16 weeks of age. ApoE KO/Gpnmb Tg mice (C57BL/6 background) were obtained by crossing ApoE KO mice with Gpnmb Tg. ApoE KO/Gpnmb Tg mice were maintained on the second HFD from 4 to 16 weeks of age and analyzed at 16 weeks of age. The animals were euthanized by intraperitoneal barbiturate injection, and tissues were quickly collected for further analyses.

### Histological examination

SA-β-gal activity was examined as described previously^9^. Briefly, cells were fixed with 0.25% Glutaraldehyde in PBS at room temperature for 15 min, then washed with PBS 1 time, followed by incubation for 1–2 h at 37 °C in β-gal staining solution containing 1 mg ml^–1^ 5-bromo-4 chloro-3-indolyl-β-D-galactoside (X-gal, Takara), 5 mmol l^–1^ potassium ferrocyanide, 5 mmol l^–1^ potassium ferricyanide, 150 mmol l^–1^ NaCl, 2 mmol l^–1^ MgCl_2_, 0.01% sodium deoxycholate, and 0.02% Nonidet P-40. Then the stained cells were taken 5 photomicrographs with BZ-9000 or BZ-X810 (Keyence). Quantification of SA-β-gal activity was performed with the ImageJ program (version 1.53a). Atherosclerotic plaque was estimated by assessing sections with Oil Red O staining. Briefly, whole aortas were dissected to remove adventitial fat, opened, and pinned flat for fixing in 4% paraformaldehyde for 12 h at room temperature. Then the pinned aortas were washed for 1 min with 60% isopropyl alcohol and incubated in 0.5% Oil Red O solution (Sigma-Aldrich) in 60% isopropyl alcohol) for 15 min at 37 °C for staining. Subsequently, the samples were briefly immersed in 60% isopropyl alcohol solution and then washed with double distilled water. After the Oil Red O-stained specimens were photographed, quantification of the plaque area was done with ImageJ.

### Physiological analyses

Mice were housed individually to monitor their health status. For the vascular reactivity assay, mice were placed on the HFD (CLEA) for 8 weeks (from 4 to 12 weeks of age). Then mice were anesthetized and euthanized, after which the iliac arteries were removed and mounted on a pressure myograph (DMT, Arhus, Denmark) for measurement of vascular reactivity as described previously^51^. The isolated vessels were perfused with physiological saline (PSS; aerated with 95% O_2_ and 5% CO_2_ to maintain a pH of 7.4) at a pressure of 100 mmHg and a temperature of 37 °C. PSS had the following composition: 118.99 mmol l^–1^ NaCl, 4.69 mmol l^–1^ KCl, 1.17 mmol l^–1^ MgSO_4_, 25 mmol l^–1^ NaHCO_3_, 2.5 mmol l^–1^ CaCl_2_, 1.18 mmol l^–1^ KH_2_PO_4_, 0.03 mmol l^–1^ EDTA, and 5.5 mmol l^–1^ glucose. Vessels were equilibrated for 15–30 min, followed by constriction with 10^–5^ M phenylephrine. Then concentration–response curves were generated by stepwise addition to the organ bath of increasing concentrations of acetylcholine (Ach; 10^–9^ to 10^–5^ mol l^–1^). Subsequently, the vessels were washed with PSS and constriction with 10^–5^ mol l^–1^ phenylephrine was repeated. Basal NO production was inhibited by addition of l-NAME at 3 × 10^–4^ mol l^–1^ for 30 min. Then concentration–response curves were generated for sodium nitroprusside (SNP) by stepwise addition of increasing concentrations from 10^–9^ to 10^–5^ mol l^–1^. Vasorelaxation responses to Ach and SNP were expressed as the percent change of force (mN). To create a hind limb ischemia model, mice were anesthetized for ligation and stripping out of the proximal femoral artery and distal popliteal artery after dissection of all side branches, as described previously^52^. Recovery of blood flow was assessed up to 28 days after surgery by measuring hind limb perfusion with a laser Doppler perfusion analyzer (Moor Instruments) after the mice were anesthetized with 0.5% isoflurane in O_2_ at 3 L min^–1^.

### Cell culture

Human umbilical vein endothelial cells (HUVECs) and human aortic endothelial cells (HAECs) were purchased from Lonza and cultured according to the manufacturer’s instructions. Replicative senescent cells were identified as cells in cultures that did not show an increase of cell numbers and remained subconfluent for 2 weeks. We also confirmed their senescent state by measuring SA-β-gal activity as well as CDKN2A and CDKN1A expression. To induce premature senescence, HUVECs and HAECs were exposed to 100 nM of doxorubicin (Doxo) for 72 h twice, followed by the incubation without Doxo treatment for 7 days. Senescence state was confirmed by increased SA-β-gal activity as well as up-regulation of CDKN2A and CDKN1A expression. Cell proliferation was also monitored as described in the next section. To induce lysosomal stress, HUVECs were treated with 100 nmol l^–1^ bafilomycin A1 (AdipoGen) for 24 h. Mouse preadipocytes were isolated and cultured as described^53^. Human melanoma cells (SK-MEL-2) were purchased from ATCC and cultured according to the manufacturer’s instructions.

### Cell proliferation

For doxorubicin-induced premature senescent cells, cells were resuspended at 2.5 × 10^3^ cells per well to a 96-well black wall/clear bottom plate (Corning) with the culture medium containing 1/500 volume of Incucyte Nuclight Rapid Red Dye (Sartorius). The cells were incubated and stained in Incucyte SX5 (Sartorius) placed in a 37 °C/5% CO_2_ incubator for 6 h, then 5 photomicrographs per well were acquired every 3 h for following 48 h. Nuclight-positive nuclei per image were counted by Incucyte software (version 2021A).

### In vitro fluorescent imaging analysis

Lysosomes were detected with LysoTracker Green DND-26 (Invitrogen) and LysoSensor green DND-189 (Invitrogen) according to the manufacturer’s directions. Lysosomal enzyme activity was analyzed by using a Lysosomal Intracellular Activity Assay Kit (K448-50; BioVision). Mitophagy was evaluated with a Mitophagy detection kit (Do-Jin-do) after 8 h of starvation according to the instructions of the manufacturer. MitoSOX Red (Invitrogen) and MitoTracker Green (Invitrogen) were used according to the manufacturer’s instruction. Stained sections were photographed with a Biorevo (Keyence Co) or FV1200 (Olympus) and fluorescence signals were quantified with ImageJ software. To detect the cellular localization of GPNMB in human melanoma cells, live cells were stained with AF555 conjugated anti-GPNMB antibody (1:100, bs-2684R A555, Bioss) or AF555 conjugated control IgG (1:100, bs-0295D A555, Bioss) for 30 min on ice. After washing, cells were incubated at 37 °C or on ice for 1 h. Then, the cells were incubated with a Wheat Germ Agglutinin Alexa Fluor 488 conjugate (1:10, Life Technologies, W11261) to stain the plasma membrane or with LysoTracker Green DND-26 to stain lysosomes and with Hoechst (1:1000, 33258, Invitrogen) to label nuclei.

### Electron microscopy

Human endothelial cells were fixed in 2.5% glutaraldehyde/2.0% paraformaldehyde in 0.1 mol l^–1^ cacodylate buffer. Then 50 mg of calcium chloride was added to 400 ml of fixative. Grids were prepared for electron microscopy, which was performed in the electron microscope core facility at Niigata University using a JEM1400 transmission electron microscope. For immunoelectron microscopy HUVECs were collected and fixed in 2% PLP (Periodate Lysine Paraformaldehyde) solution (Wako 290–63201) at 4 °C overnight and washed with PBS for 3 times. Immunogold labeling was carried out using anti-rabbit IgG (H + L) 15 nm Gold (BBI Solutions EM.GAR15) antibody.

### Viral infection and transfection

Small-interfering RNAs (siRNAs) for *GPNMB, MITF, TFEB, TFE3,* or *TFEC* were purchased from Ambion and introduced into pre-senescent or senescent HUVECs by using Lipofectamine RNAiMax (Invitrogen) according to the manufacturer’s instructions. HUVECs were analyzed at 72 h after transduction. In some experiments, siRNA-transduced HUVECs were deprived of serum and amino acids for 8 h before analysis. We purchased a plasmid encoding full-length human *GPNMB* from DNAFORM and generated human GPNMB-expressing retroviral vectors using pLNCX (Clontech) and pRetroQ-mCherry-N1 (Clontech). For knockdown experiments, we used the Knockout RNAi System (Clontech), and generated a retroviral vector expressing short hairpin RNA (shRNA) targeting human *GPNMB* according to the directions of the manufacturer. We used pSIREN-RetroQ as the negative control vector. Human endothelial cells (4–6 passages) were plated in 100-mm diameter dishes at 4–5 × 10^5^ cells at 24 h before infection, after which the culture medium was replaced by retroviral stock supplemented with 8 mg ml^–1^ polybrene (Sigma). From 48 h after infection, cells were selected by culture for 7 days in 500 mg ml^–1^ G418 (pLNCX-based vectors) or 0.5 mg ml^–1^ puromycin (pRetroQ-mCherry-based or pSIREN-RetroQ-based vectors). Then 4 × 10^5^ selected cells were seeded in 100-mm diameter dishes on the 8th day after infection, which was designated as day 0.

### RNA analysis

Total RNA (1 μg) was isolated from tissue samples with RNA-Bee (TEL-TEST Inc.) Real-time quantitative PCR (qPCR) was performed by using a Light Cycler 480 (Roche) with the Universal Probe Library and Light Cycler 480 Probes Master (Roche) according to the manufacturer’s directions. The primers used and their sequences were as follows (*Rplp0, RPLP0* or *ACTB* was used as the internal control).

Mouse primers:

*Gpnmb; 5′- ACGGCAGGTGGAAGGACT -3′, 5′- CGGTGAGTCACTGGTCAGG -3′*

*Cdkn1a; 5′-TCCACAGCGATATCCAGACA -3′, 5′-GGACATCACCAGGATTGGAC -3′*

*Cdkn2a; 5′-GGGTTTTCTTGGTGAAGTTCG -3′, 5′-TTGCCCATCATCATCACCT -3′*

*Rplp0; 5′- GATGCCCAGGGAAGACAG -3′, 5′- ACAATGAAGCATTTTGGATAA -3′*

Human primers:

*GPNMB*; 5*′*- ACCCACCCCTTCTTTAGGAC -3*′*, 5*′*- TCTGGCAGTTTTCATCAGGA -3*′*

*CDKN1A*; 5*′*- TCACTGTCTTGTACCCTTGTGC -3*′*, 5*′*- GGCGTTTGGAGTGGTAGAAA -3*′*

*CDKN2A*; 5*′*- GTGGACCTGGCTGAGGAG -3*′*, 5*′*- CTTTCAATCGGGGATGTCTG -3*′*

*MITF*; 5*′*- CAAAAGTCAACCGCTGAAGA -3*′*, 5*′*- AGGAGCTTATCGGAGGCTTG -3*′*

*TFEB*; 5*′*-CGGCAGTGCCTGGTACAT -3*′*, 5*′*- CTGCATGCGCAACCCTAT -3*′*

*TFEC*; 5*′*-CGGTATGGAATCAAGTTTTAAAGAG -3*′*, 5*′*-TCACCGCTATACACATCCAAAA -3*′*

*TFE3*; 5*′*-CCTTACCCCTTCGCTCAAG -3*′*, 5*′*- TGCAGAAGACGATGCAGAGA -3*′*

*RPLP0*; 5*′*- TCTACAACCCTGAAGTGCTTGAT -3*′*, 5*′*- CAATCTGCAGACAGACACTGG -3*′*

*ACTB*; 5*′*- CCAACCGCGAGAAGATGA -3*′*, 5*′*- CCAGAGGCGTACAGGGATAG -3*′*

### RNA sequencing analysis

Total RNA was extracted from young and senescent HUVECs or aorta samples obtained from ApoE KO mice treated with Gpnmb or control vaccination using the RNeasy Plus Micro Kit (Qiagen). The cDNA libraries were generated using the NEBNext Ultra RNA Library Prep Kit (New England BioLabs, Beverly, MA, USA). The quality of total RNA and cDNA were assessed using the Agilent 2100 Bioanalyzer with the RNA6000 nano Kit and the DNA7500 kit (Agilent Technologies). Sequencing was performed using the HiSeq1500 system (Illumina) with a single-read sequencing length of 60 bp. A quality check of the sequencing data was performed by FaQCs (version 1.34). The TopHat analysis (version 2.0.13) was used to map the reference genome (GRCh38/hg38), with annotation data downloaded using the Ensembl Asia website (URL https://asia.ensembl.org/). Expression of each transcript was quantified as number of fragments/kilobase of transcript/per million fragments mapped (FPKM). Expression of each transcript was compared between the 2 groups (young and senescent HUVECs or Gpnmb and control vaccine) via the Cuffdiff program (version 2.2.1). Gene expression data obtained in these studies were deposited in the Gene Expression Omnibus database (GSE155680 for HUVECs).

### Western blot analysis

Whole-cell lysates were prepared in lysis buffer (10 mmol l^–1^ Tris–HCl, pH 8, 140 mmol l^–1^ NaCl, 5 mmol l^–1^ EDTA, 0.025% NaN_3_, 1% Triton X-100, 1% deoxycholate, 0.1% SDS, 1 mmol l^–1^ PMSF, 5 μg ml^–1^ leupeptin, 2 μg ml^–1^ aprotinin, 50 mmol l^–1^ NaF, and 1 mmol l^–1^ Na_2_VO_3_). Then lysates (40–50 μg) were resolved by SDS-PAGE and proteins were transferred to PVDF membranes (Millipore), which were incubated with the primary antibody followed by incubation with horseradish peroxidase-conjugated anti-rabbit or anti-mouse immunoglobulin-G (Jackson). Specific proteins were detected by enhanced chemiluminescence (Amersham). GPNMB was immunoprecipitated from lysates of young and senescent HUVEC with anti-GPNMB antibody (#13251, Cell Signaling) according to the manufacturer’s recommendations, and normal rabbit IgG (#3900, Cell Signaling) was used as the control. The primary antibodies for Western blotting were anti-p53 antibody (2524; Cell Signaling), anti-p21 antibody (ab7960; Abcam), anti-Gpnmb antibody (AF2330; R&D Systems), anti-actin antibody (4967; Cell Signaling), and anti-GAPDH antibody (sc-20357; Santa Cruz). For studies of human vascular endothelial cells, anti-p53 antibody (DO1) (sc-126; Santa Cruz), anti-p21 antibody (OP64-20UG; Calbiochem), anti-p16 antibody (554079; BD) anti-ATP6V1A antibody (ab199326; Abcam), and anti-GPNMB antibody (13251; Cell Signaling) were used. All primary antibodies were employed at a dilution of 1:1000, except for anti-actin antibody and anti-GAPDH antibody (1:5000). The secondary antibody for anti-p53 antibody (2524; Cell Signaling), anti-p21 antibody (OP64-20UG; Calbiochem), and anti-p16 antibody (554079; BD) was peroxidase-conjugated AffiniPure goat anti-mouse IgG (light chain specific) (115–035-174; Jackson ImmunoResearch). In addition, peroxidase-conjugated AffiniPure goat anti-mouse IgG (H + L) (115-035-003; Jackson ImmunoResearch) was the secondary antibody for anti-p53 antibody (DO1) (sc-126), while peroxidase-conjugated AffiniPure goat anti-rabbit IgG (H + L) (111–035-003; Jackson ImmunoResearch) was used for anti-GPNMB antibody (13251), anti-actin antibody (4967), anti-ATP6V1A antibody, and anti-p21 antibody (ab7960), and peroxidase-conjugated AffiniPure donkey anti-goat IgG (H + L) (705–035-147; Jackson ImmunoResearch) was employed for anti-GAPDH antibody (sc-20357) and anti-Gpnmb antibody. All secondary antibodies were added at a dilution of 1:10000. For immunoprecipitation, anti-GPNMB antibody (#13251; Cell Signaling) was used according to the manufacturer’s instructions. Full Western blot images are shown in the Supplementary Data.

### Chromatin immunoprecipitation assay

Chromatin immunoprecipitation (ChIP) was performed with chromatin prepared from young and irradiation-induced senescent HUVECs. Senescence was induced by 10 Gy of cesium radiation. We confirmed these cells to become senescent at 10 days after irradiation. Sonicated chromatin was immunoprecipitated by the antibody against TFEB (#37785, Cell Signaling) or TFEC (ab93808, Abcam), and the precipitates were collected with Dynabeads protein G (Life technologies). Real-time PCR targeting the putative MITF/TFEs binding element in the *GPNMB* promoter region was performed with the following pair of primers.

Forward: 5′- CAGTGCCGCTTAATACCATCAC -3′.

Reverse: 5′- GCAACAGTGTTCTTCTGGCATC -3′.

### ATAC-seq

ATAC-seq was performed as described previously^54^. Briefly, the nuclei were isolated from 50,000 cells and transposed by incubation with Omni-ATAC transposition mix for 30 min at 37 °C in a thermomixer with shaking at 1,000 r.p.m. Reactions were cleaned up with Zymo DNA Clean and Concentrator-5 kit. Pre-amplification of purified DNA was done with 5 PCR cycles, while the number of cycles for final amplification was determined by qPCR, as described previously^55^. After purification using the MinElute PCR Purification Kit (Qiagen), the libraries were quantified with Bioanalyzer (Agilent). Then 50 bp single read sequencing was performed using an Illumina HiSeq3000 instrument. Analysis of ATAC-seq data was done with TrimGalore v0.5.0, BWA v0.7.17, MACS v1.4.3, and Picard v2.18.20. First, trimming of Nextera adaptor sequences and quality trimming of the reads were performed using TrimGalore with the following options: “–quality 20 –phred33 –stringency 3 –gzip –length 20 –nextera –trim1”. These reads were aligned to the human reference genome hg19 by using BWA MEM with the default parameters^56^. Then the aligned read were employed for peak calling by MACS with the default parameters^57^. After removal of duplicate reads using Picard (http://broadinstitute.github.io/picard/), the aligned reads and called peaks were visualized on the Integrative Genomics Viewer^58^.

### Proteomic analysis

Cells stably expressing mCherry or GPNMB-mCherry were lysed by sonication (3 × 5 s-bursts) on ice in Pierce IP lysis buffer (25 mmol l^–1^ Tris–HCl pH 7.4, 150 mmol l^–1^ NaCl, 1 mmol l^–1^ EDTA, 1% NP-40, and 5% glycerol). The lysates were subjected to immunoprecipitation with anti-mCherry antibody (ab213511; Abcam), and the precipitates were transferred to a new microcentrifuge tube for “tube gel digestion”, a modified in-gel digestion procedure^59^, to generate dithiothreitol-reduced, iodoacetamide-alkylated tryptic peptides for mass spectrometry. Briefly, an aliquot of each immunoprecipitate (30 μl) was mixed with 15 μl of acrylamide (40% T, acrylamide : *N*, *N*’-methylene bis(acrylamide) at 9:1 by weight) and 0.5 μl each of 10% ammonium persulfate and *N*, *N*, *N*′, *N*′-tetramethylenethylenediamine, followed by polymerization for 60 min at room temperature. The polymerized tube gels were fixed in 50% methanol with 7% acetic acid for 30 min, and kept in 5% acetic acid at 4 °C. Then the tube gels were subjected to in-gel digestion with trypsin (Sigma, Proteomics sequencing grade) by essentially the same method as described previously^60^.

Peptides from each digested gel were dissolved in 15 μl of 0.3% formic acid, and aliquots (3 μl) were injected into a nano-flow liquid chromatograph (Eksigent nanlLC 415 with ekspert cHiPLC, Sciex) that was coupled with a tandem mass spectrometer (TripleTOF5600 + , Sciex) via a nano-ESI ion source. Analysis of each sample was conducted in duplicate in the trap and elute modes, using a ChromeXP C18 Chip column (200 μm × 0.5 mm) as the trap column and a spray needle C18 column (75 μm × 150 mm) provided by Nikkyo Technos as the analytical column. Mobile phases A and B were 0.1% formic acid and 0.1% formic acid in acetonitrile, respectively. Peptides were eluted with a gradient from 2 to 32% of mobile phase B over 40 min at 300 nl minute^–1^. The MS spectrum (250 ms) and 10 MS/MS spectra (100 ms each) were acquired in the data-dependent mode. The dynamic exclusion time was set at 5 s. Autocalibration was performed every 4 samples using 50 fmol of bovine serum albumin tryptic peptides as calibrant (KYA technology).

### Bioinformatic analysis

Gene expression profiles for GSE37091 and GSE13712 were analyzed with the GEO2R analysis tool provided as a web service in the Gene Expression Omnibus database^61^. Genes showing three-fold or greater upregulation in senescent samples in comparison with young samples were extracted. The presence or absence of transmembrane regions was predicted with TMHMM2.0 for protein sequences downloaded from the KEGG GENES database (May 2, 2014 version)^62,63^, and this process identified ten genes with predicted transmembrane regions. From among them, we selected *GPNMB* as a candidate seno-antigen gene because there is evidence of a potential link between *GPNMB* and human aging^64^. Proteomics data were analyzed by using DAVID and STRING. For GO enrichment analysis, we excluded molecules with GO terms that included “RNA binding” because of possible nonspecific binding to GPNMB. Data were submitted to jPOST (JPST000594)^65^.

### Statistical analysis

Statistical analysis was done with SPSS software (version 24 or 25). All data were from different biological replicates and are shown as box and whisker plots that display the range of the data (whiskers), the 25th and 75th percentiles (box), and the median (solid line) or the mean ± SEM or SD. Outliers and abnormal values were assessed by boxplot analysis. Differences between groups were examined by the two-tailed Student’s *t* test, one-way ANOVA or two-way ANOVA followed by Tukey’s multiple comparison test for comparisons among more than two groups, or repeated measures ANOVA analysis followed by Tukey’s multiple comparison test was done for time course comparisons. In all analyses, P < 0.05 was considered to indicate statistical significance.

## Supplementary Information

Supplementary Information 1.

Supplementary Information 2.
