# Supplementary material for: Glycoprotein nonmetastatic melanoma protein B regulates lysosomal integrity and lifespan of senescent cells
Source: Sci Rep. 2022 Apr 20;12:6522. doi: 10.1038/s41598-022-10522-3 (PMC9021310; doi:10.1038/s41598-022-10522-3)
Supplement: Supplementary file 2 — Supplementary Information 2. [file 41598_2022_10522_MOESM2_ESM.pdf]

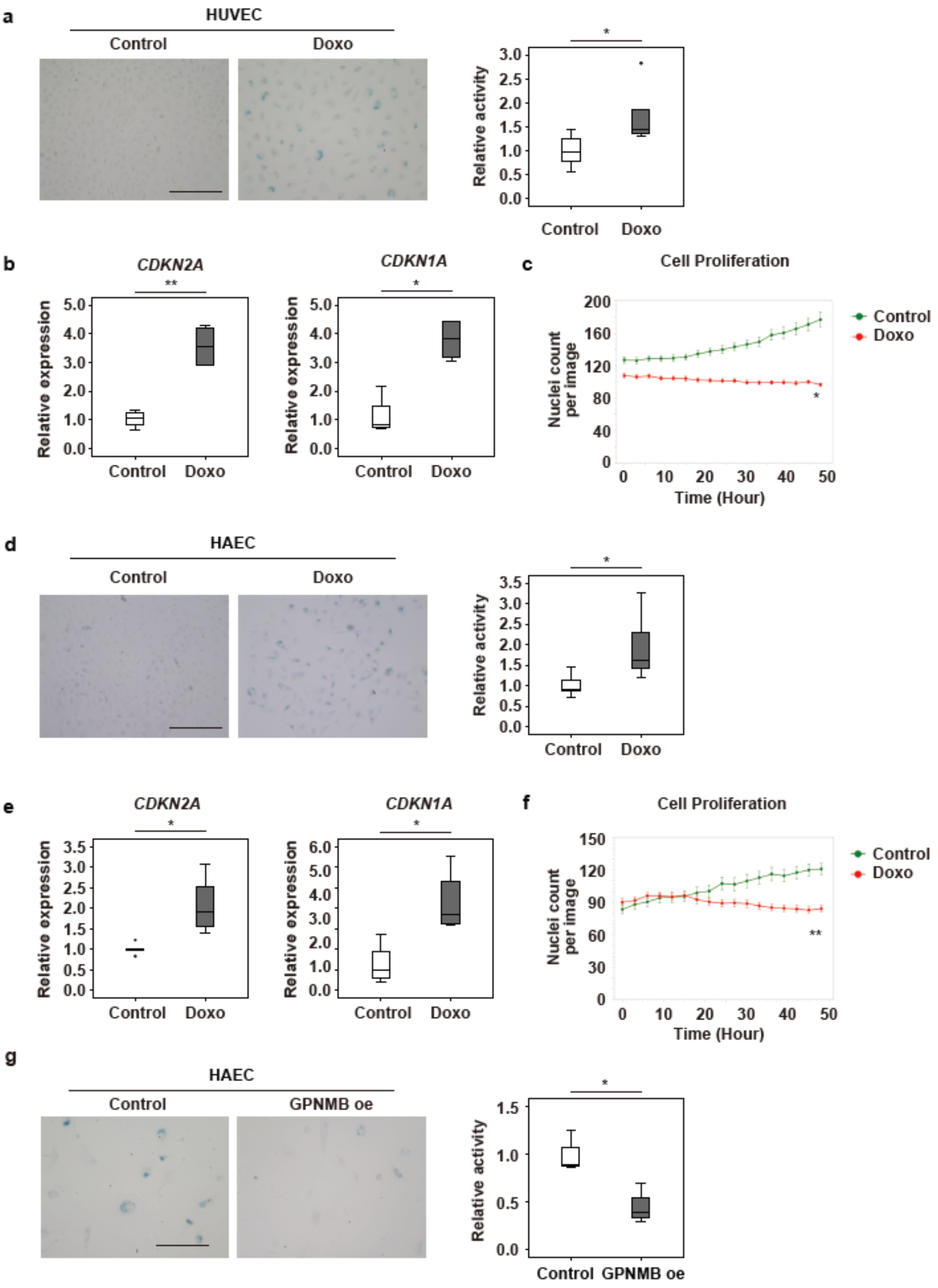


**Supplementary Figure 1. Doxorubicin-induced premature senescence.**
**a-c,** HUVECs were treated with 100 nM of doxorubicin (Doxo) for 72 hours twice, followed by the incubation without treatment for 7 days. SA-β-gal activity (**a**, n=6, 6), expression of *CDKN2A* and *CDKN1A* (**b**, n=4, 4), and cell proliferation (**c**, n=6, 6) were examined. **d-f,** Human aortic endothelial cells (HAECs) were treated with 100 nM of doxorubicin as indicated in Supplementary Fig. 1a. SA-β-gal activity (**d**, n=6, 6), expression of *CDKN2A* and *CDKN1A* (**e**, n=5, 5), and cell proliferation (**f**, n=6, 6) were examined. **g,** SA-β-gal assay of HAECs infected with a GPNMB overexpression vector

(GPNMB oe) or a control vector (Control) after treatment with doxorubicin as indicated in Supplementary Fig. 1a (n=3, 3). Data were analyzed by the two-tailed Student's t-test (**a, b, d, e, g**) or by repeated measures ANOVA analysis (**c, f**). *P<0.05, **P<0.01. The data are shown as box and whisker plots (**a, b, d, e, g**) or the mean ± SD (**c, f**). Scale bar=200 $\mu$ m.

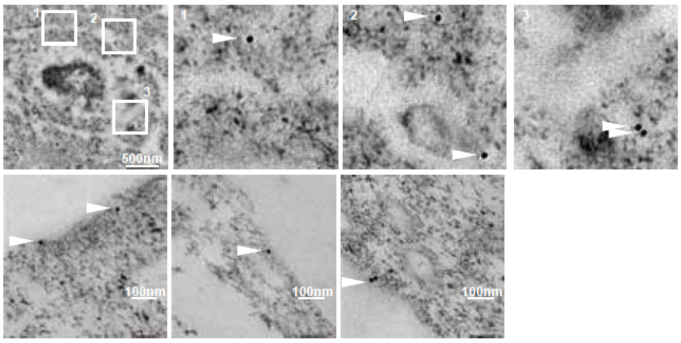


**Supplementary Figure 2. Immunoelectron microscopy.**
Immunoelectron microscopy labeling mCherry (arrow heads) in HUVECs overexpressing GPNMB-mCherry. GPNMB-mCherry was localized in the lysosomes (upper panels) as well as in the plasma membrane (lower panels).

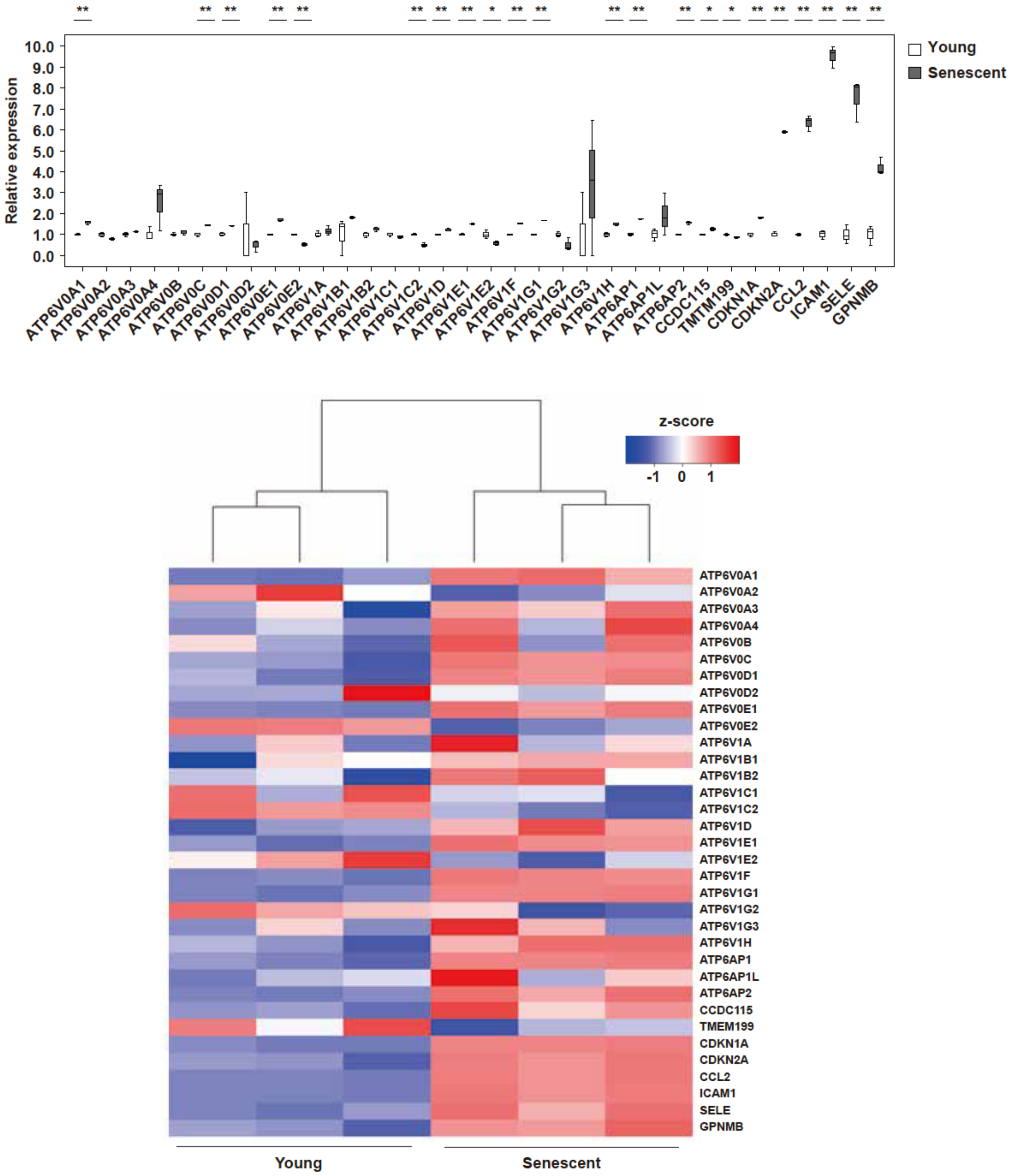


**Supplementary Figure 3. Expression of V-type ATPase components in young and senescent endothelial cells.**

Young and senescent HUVECs underwent RNA sequencing analysis. The expression of V-type ATPase components along with senescence markers is shown (n=3, respectively). The data were analyzed by the two-tailed Student's t-test and are presented as box and whisker plots. *P<0.05, **P<0.01.

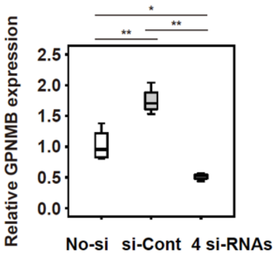


**Supplementary Figure 4. Expression of Gpnmb after introduction of siRNAs.**
Relative expression of *GPNMB* in replicative senescent HUVECs after introduction of siRNAs for the MITF/TFE transcription factors (4 si-RNAs: si-MITF, si-TFEB, si-TFEC, and si-TFE3), control siRNA (si-Cont), or no siRNA (No-si) (n=4, 4, 3). Data were analyzed by two-way ANOVA analysis followed by Tukey's multiple comparison. *P<0.05, **P<0.01. The data are shown as box and whisker plots.

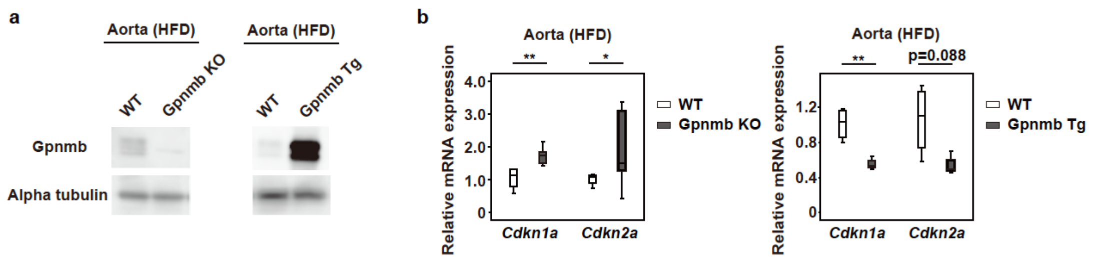


**Supplementary Figure 5. Expression of Gpnmb and senescence markers in Gpnmb KO/Tg mice.**

**a**, Western blot analysis for expression of Gpnmb in the aortas of Gpnmb KO/Tg mice or littermate controls (WT) on the HFD. Original blots are presented in Supplementary Figure 10. **b**, qPCR for relative expression of *Cdkn1a* and *Cdkn2a* in the aortas of Gpnmb KO/Tg mice or littermate controls (WT) on the HFD (n=4 for WT and n=8 for KO, n=4 for WT and n=3 for Tg). The data were analyzed by the two-tailed Student's t-test (**b**). **P<0.01. The data are shown as box and whisker plots (**b**).

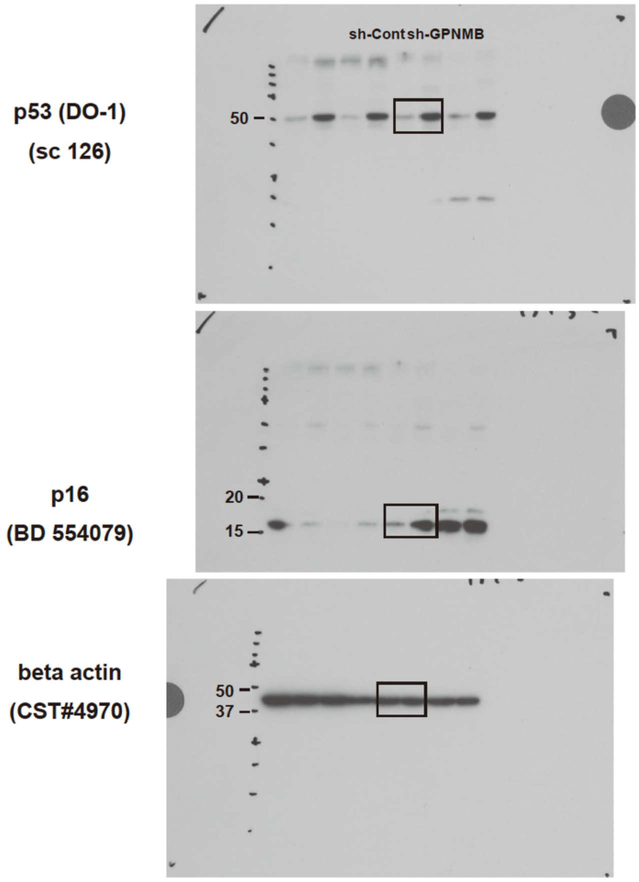


**Supplementary Figure 6. Full blots of Figure 1b**

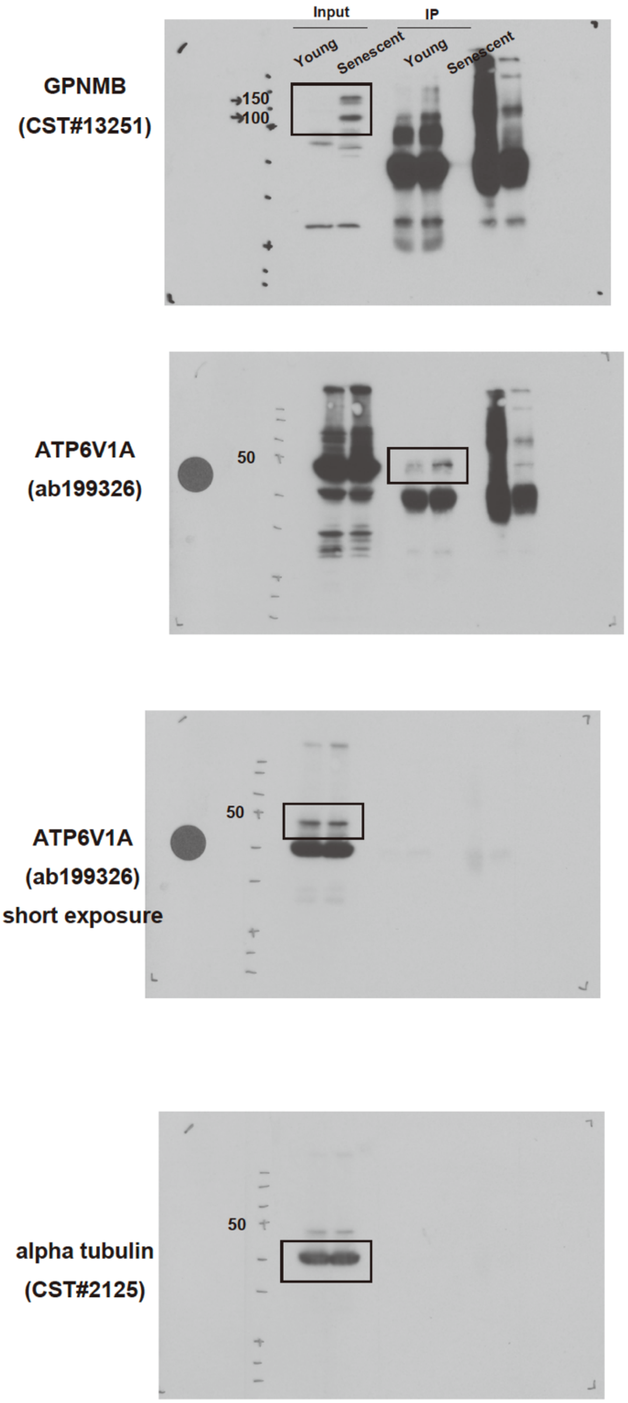


**Supplementary Figure 7. Full blots of Figure 3b**

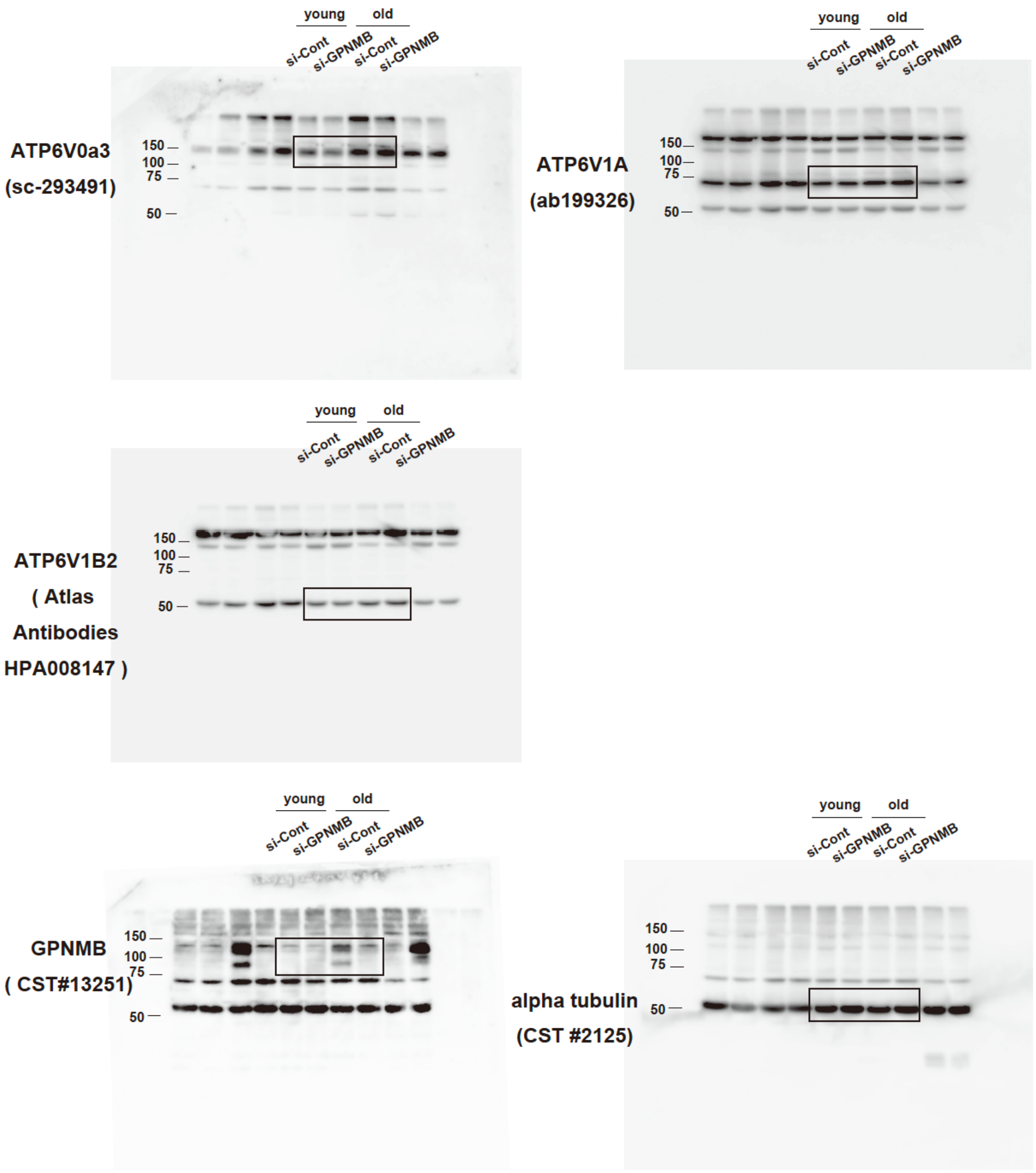


**Supplementary Figure 8. Full blots of Figure 3c**

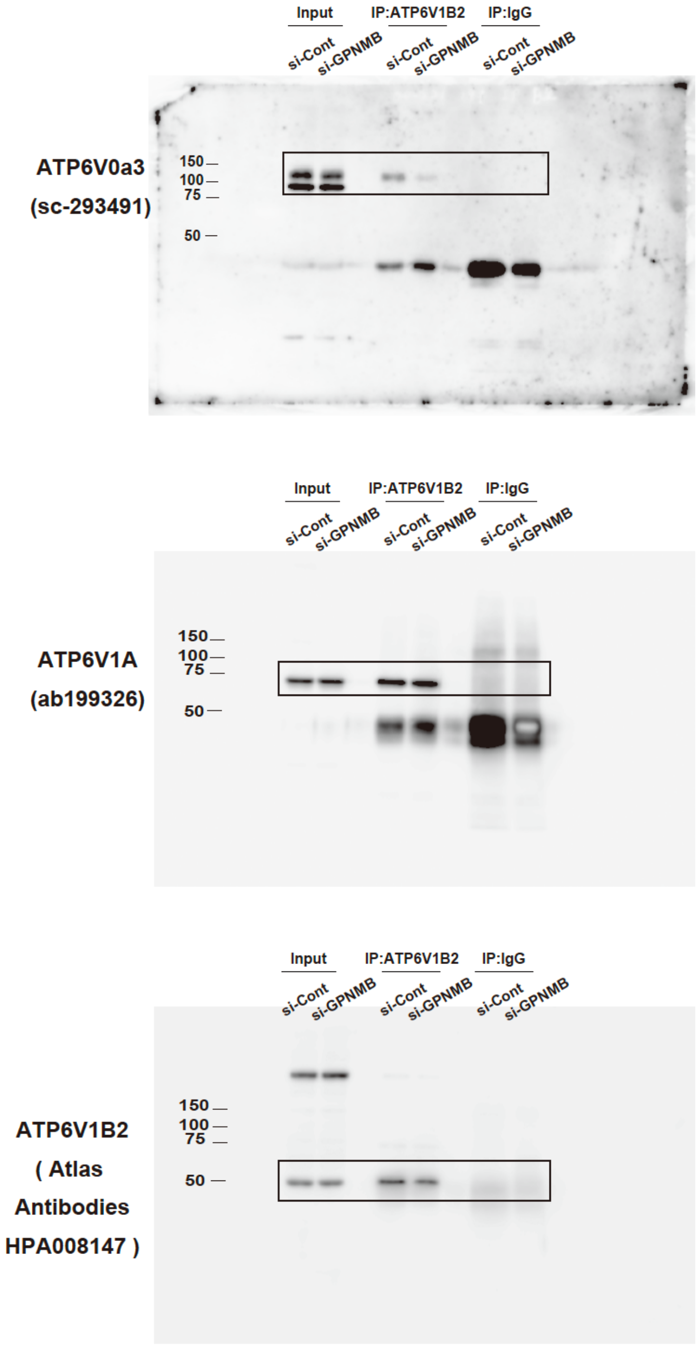


**Supplementary Figure 9. Full blots of Figure 3d**

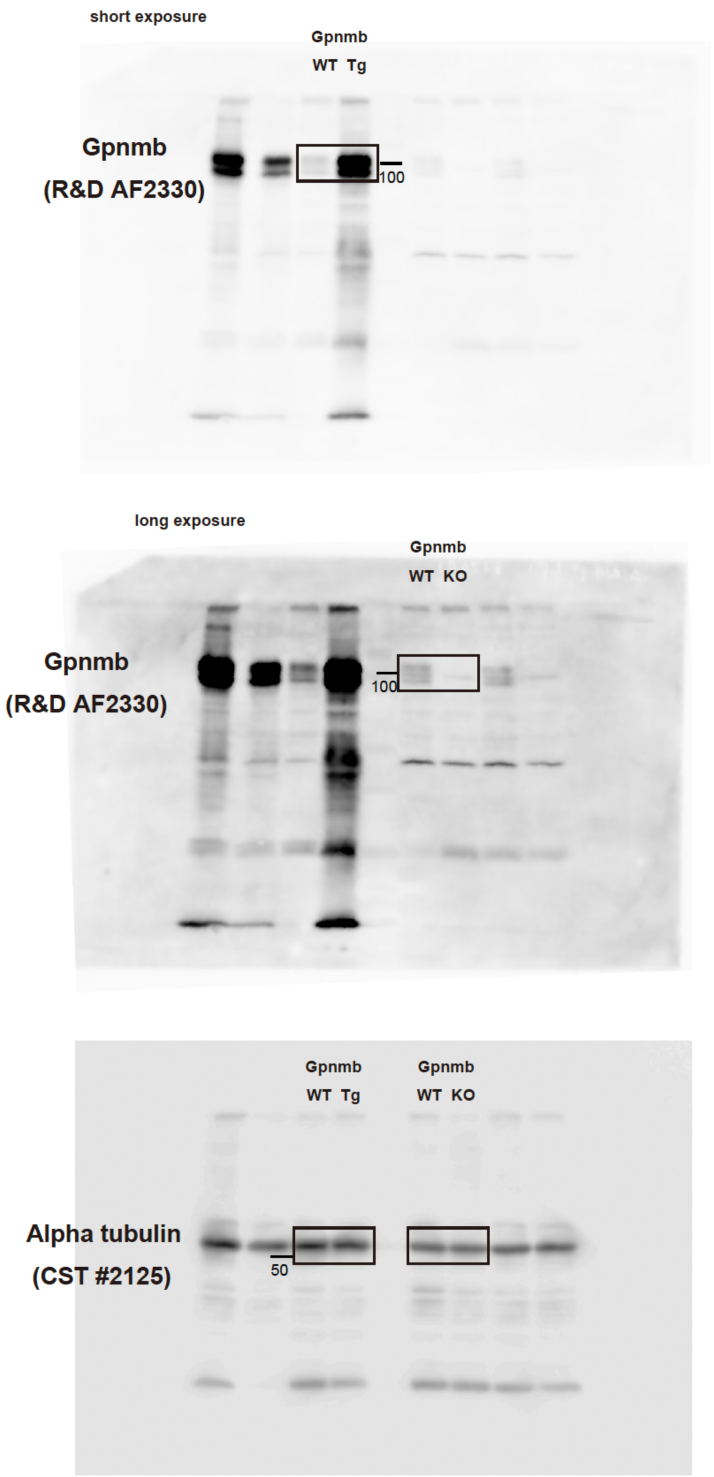


**Supplementary Figure 10. Full blots of Supplementary Figure 5a**
